# A new species of *Dictyochaeta* (Sordariomycetes, Chaetosphaeriales, Chaetosphaeriaceae) from freshwater habitats in China

**DOI:** 10.3897/BDJ.11.e97439

**Published:** 2023-04-28

**Authors:** Xin-Yi Yan, Jun-En Huang, Hai-Yan Song, Yang Gao, Hai-Jing Hu, Zhi-Jun Zhai, Jun-Qing Yan, Guang-Hua Huo, Dian-Ming Hu

**Affiliations:** 1 Jiangxi Key Laboratory for Conservation and Utilization of Fungal Resources, Jiangxi Agricultural University, Nanchang, China Jiangxi Key Laboratory for Conservation and Utilization of Fungal Resources, Jiangxi Agricultural University Nanchang China; 2 Jiangxi Agricultural University, Nanchang, China Jiangxi Agricultural University Nanchang China; 3 Bioengineering and Technological Research Centre for Edible and Medicinal Fungi, Jiangxi Agricultural University, Nanchang, China Bioengineering and Technological Research Centre for Edible and Medicinal Fungi, Jiangxi Agricultural University Nanchang China; 4 Chinese Academy of Sciences, Beijing, China Chinese Academy of Sciences Beijing China; 5 Key Laboratory of Crop Physiology, Ecology and Genetic Breeding, (Jiangxi Agricultural University), Ministry of Education of the P.R., Nanchang, China Key Laboratory of Crop Physiology, Ecology and Genetic Breeding, (Jiangxi Agricultural University), Ministry of Education of the P.R. Nanchang China

**Keywords:** dematiaceous hyphomycete, new species, taxonomy, phylogeny

## Abstract

**Background:**

Freshwater fungi refer to the fungi that depend on the freshwater habitats for the whole life cycle or part of their life cycle. In this context, a new aquatic hyphomycete was isolated from decaying wood in a freshwater habitat in Jiangxi Province, China.

**New information:**

*Dictyochaetajiangxiensis* sp. nov., a new aquatic hyphomycete, is characterised by its unbranched, septate, base-fertile conidiophores with multisepta and single phialide at the apex, brown, sterile seta, monophialidic, subcylindrical conidiogenous cells narrowing below the funnel-shaped collarette, hyaline, unicellular, thin-walled, smooth, guttulate, falcate to subclavate conidia narrowly rounded at both ends with hair-like appendages. Phylogenetically, the new species *Dictyochaetajiangxiensis* clustered together with *Dictyochaetabrevis* MFLU 19-0216 in a well-supported clade, but formed a separate branch. In order to better define the taxonomic status of the new species, a phylogenetic tree of most closely-related taxa in Chaetosphaeriaceae was established, based on multi-locus sequences (ITS and LSU). The novel species is described and illustrated. Newly-generated molecular data of *Dictyochaetajiangxiensis* is also provided.

## Introduction

Spegazzini (1923) established the genus *Dictyochaeta* with *D.fuegiana* as type species, which was isolated from fallen leaves of *Nothofagusbetuloides* (Mirb.) Oerst. Gamundí et al. (1977) and Godeas et al. (1977) verified the holotype and redescribed it. Later, Réblová (2004) re-examined the type species and gave more detailed description about the genus *Dictyochaeta*, as two-layer conidiophores, the upper layer setiform, when sterile, monophialidic or rarely polyphialidic, the lower layer always fertile, monophialidic, rarely polyphialidic, collarette on conidiogenous cells and aseptate, hyaline, falcate conidia without setulae. Since then, more and more species have been discovered and classified as or transferred to *Dictyochaeta* and its molecular and morphological data have been expanded. According to literature and herbarium records, the *Dictyochaeta*-like fungi are globally distributed in the Holarctic Region and the Tropics and grow on decaying plant, such as bark, wood, bamboo culms, palm fronds, fallen leaves and petioles in freshwater and terrestrial environments. They also occur as plant pathogens or endophytes in living plants. (Agnihothrudu 1968, Lunghini et al. 1971, Shearer and Crane 1971, Sutton and Hodges 1975, Hewings and Crane 1981, Holubová-Jechová 1984, Kuthubutheen 1987, Kuthubutheen and Nawawi 1990, Kuthubutheen and Nawawi 1991a, Kuthubutheen and Nawawi 1991b, Réblová et al. 1999, Kirschner and Chen 2002, Crous et al. 2014, Crous et al. 2015, Maharachchikumbura et al. 2016).

There are always different opinions on the classification of *Dictyochaeta*, which is considered as a synonym of several genera, such as *Codinaea* Maire., *Menispora* Pers. and *Menisporopsis* S. Hughes. Previously, *Dictyochaeta* was usuallly regarded as a synonym of *Codinaea* (Maire 1937), because they share greatly similar features on phialidic conidiogenous cells and setae, but differ mainly in the conidia without setulae (Cai et al. 2006). Crous et al. (2018) thought that priority should be given to the older name, *Dictyochaeta*. Réblová (2000) recommended that species with setulae should be classified into *Codinaea* and those without setulae into *Dictyochaeta*. Réblová et al. (2021a) re-evaluated the concept of *Dictyochaeta* and revised species delimitation, based on six loci (ITS, LSU, SSU, RPB2, TEF1-α, TUB2) along with comparative morphological and cultivation studies. In their study, some species of *Dictyochaeta*, such as *D.siamensis*, *D.simplex* etc. clustered within the clade *Codinaea* with a high support. As for the demarcation between *Dictyochaeta* and *Codinaea*, Réblová et al. (2021a) supported using conidial appendages as a classification criterion to distinguish *Dictyochaeta* from *Codinaea* (Réblová and Winka 2000). Based on revised species, morphological characteristics of conidia (shape, septation, absence or presence of setulae), collarettes (shape) and setae (presence or absence) and extension of the conidiogenous cell proved to be important at the generic level. To date, *Dictyochaeta*-like fungi, together with *Codinaea*-like fungi, were divided into five lineages in the phylogenetic analyses (Réblová et al. 2021b). Dual DNA barcoding and ancestral reconstruction of ecological and geographic distribution facilitated re-assessment of *Dictyochaeta*-like fungi. Réblová et al. (2021b) introduced five genera (*Codinaeella*, *Nimesporella*, *Stilbochaeta*, *Tainosphaeriella* and *Xyladelphia*) to accommodate *Codinaea*-like fungi and retained the taxonomic status of *Dictyochaeta* sensu stricto.

## Materials and methods

### Sample collection and specimen examination

Submerged wood samples were collected in a stream from Jishui County, Ji'an City, Jiangxi Province, China on 9 April 2018. The samples were taken to the laboratory in ziplock bags and placed in plastic boxes. The microscopic analysis was performed by a stereomicroscope to observe the fungal fruiting body on a natural substrate. Micro-examination and photomicrographs were taken under a compound microscope (Nikon Ni). The specimens were deposited in the Herbarium of Fungi, Jiangxi Agricultural University (HFJAU), Nanchang, China.

### DNA extraction, PCR amplification and sequencing

Cultures were grown at room temperature on potato-dextrose agar (PDA). Mycelia were directly scraped off from plates and transferred into centrifugal tube after fragmentation. DNA was extracted with the CTAB method following Doyle and Doyle (1987). Approximately 500 mg of mycelium was mixed with ca. 0.2 g of white quartz sand and ground with preheated (ca. 65°C) 2 × CTAB buffer [2% (w/v) CTAB; 100 mM Tris-HCl; 1.4 M NaCl; 20 mM EDTA, pH 8.0]. DNA was extracted by chloroform:isoamyl alcohol (24:1) and precipitated by isopropanol at -20°C. The DNA precipitation was purified by 70% ethanol to remove remaining impurities. Approximately 50-100 μl TE buffer or deionised water were added and stored at -20°C. Dried DNA was dissolved in deionised water at 37°C and stored at -20°C.

DNA amplification was performed by polymerase chain reaction (PCR). LSU, TUB2, EF1-α and ITS regions were amplified using primers LR0R and LR5 (Vilgalys and Hester 1990, Rehner and Samuels 1995), EF1-983F and EF1-2218R (van den Brink et al. 2012), ITS1 and ITS4 (White et al. 1990) and T1 & Bt2b (Glass and Donaldson 1995, O'Donnell and Cigelnik 1997) with 25 μl of the final volume including 9.5 μl ddH_2_O, 12.5 μl 2 ×Taq PCR MasterMix (Qingke, Changsha, China), 1 μl of DNA template and 1 μl of each primer (10 μM). The PCR reaction was under the following conditions: 94°C for 4 min, then 35 cycles of 94°C for 60 s, 53℃ (ITS, LSU, TEF1-a), 55℃ (RPB2) for 60 s and 72°C for 80 s, followed by a final extension step of 72°C for 8 min (Wu et al. 2014). PCR products were checked on 2% agarose electrophoresis gels stained with GelRed. DNA sequencing was performed using the primers mentioned above by Tsingke, Changsha, China.

### Phylogenetic analyses

The novel sequences and reference sequences collected from GenBank were aligned with MAFFT v.7.036 (http://mafft.cbrc.jp/alignment/server, Katoh et al. (2019)). The multilocus sequences were concatenated by PhyloSuite v.1.2.2 (Zhang et al. 2020). The concatenated aligned datasets were analysed separately using Maximum Likelihood (ML) and Bayesian Inference (BI). The best-fit models of evolution for the two loci tested were estimated by PhyloSuite v.1.2.2 (Zhang et al. 2020). The ML analyses were conducted with RAxML v.7.2.6 (Stamatakis and Alachiotis 2010) using a GTRGAMMA substitution model with 1000 bootstrap replicates. The robustness of the analyses was evaluated by bootstrap support (MLBS). Markov Chain Monte Carlo (MCMC) methods in MrBayes was used to estimate the posterior probabilities (PP) (Zhaxybayeva and Gogarten 2002). Trees were sampled every 100 generations. The MCMC sampling was set as four chains (three hot chains and one cold chain) running 2,000,000 generations simultaneously, resulting in 20001 total trees.

The first 25% of trees were discarded as burn-in trees and the remaining trees were used to calculate posterior probabilities. Posterior probabilities values of the BI analyses (BPP) over 0.95 were regarded to be important. Sequences generated in this study were displayed in GenBank (Table 1).

## Taxon treatments

### Dictyochaeta jiangxiensis

J.E. Huang, X.Y. Yan, H.Y. Song & D.M. Hu sp. nov.

0EF44D6F-1CC9-5267-BF2D-263CEA441DD0

846403

#### Materials

- **Type status:** Holotype. **Occurrence:** occurrenceID: B23E3165-B465-54BD-ADAE-FB318AEB9F70; **Taxon:** scientificName: *Dictyochaetajiangxiensis*; acceptedNameUsage: *Dictyochaetajiangxiensis* J.E. Huang, X.Y. Yan & D.M. Hu; kingdom: Fungi; phylum: Ascomycota; class: Sordariomycetes; order: Chaetosphaeriales; family: Chaetosphaeriaceae; genus: Dictyochaeta; specificEpithet: *jiangxiensis*; taxonRank: species; verbatimTaxonRank: species; scientificNameAuthorship: J.E. Huang, X.Y. Yan & D.M. Hu; **Location:** continent: Asia; country: China; stateProvince: Jiangxi Province; county: Jishui county; locality: Dingjiang; verbatimLatitude: 27.127397 N; verbatimLongitude: 115.276527 E; **Identification:** identifiedBy: J.E Huang, X.Y. Yan; **Record Level:** type: PhysicalObject; language: en; rightsHolder: Dian-Ming Hu; institutionID: HFJAU 3175; collectionID: HJ0108-1; institutionCode: the Herbarium of Fungi, Jiangxi Agricultural University (HFJAU); collectionCode: Fungi; ownerInstitutionCode: the Herbarium of Fungi, Jiangxi Agricultural University (HFJAU); basisOfRecord: PreservedSpecimen

#### Description

Saprobic on decaying submerged wood. **Sexual morph** Undetermined. **Asexual morph** Hyphomycetous. *Colonies* effuse, aggregate, spreading very widely, glistening white to transparent spots and short dark brown hairs. *Mycelium* composed of partly immersed and partly superficial, brown to dark brown, septate. *Setae* of the upper layer sterile, brown to black, usually associated with the conidiophores and together these can form small clusters originating from a knot of superficial hyphae, 200–420 × 4.6–7.2 μm, cylindrical, straight or slightly flexuous, septate, smooth, thick-walled, base swollen 9–12 μm wide, tapering to terminal. *Conidiophores* of the lower layer always fertile, mononematous, macronematous, erect or flexuous, unbranched, 26–60 × 3.5–5 μm (av. = 48.3 × 4.0 µm, n = 20), 3–8-septate, smooth, thin-walled, base brown 4.8–7.5 µm, apex pale brown with single phialide. *Conidiogenous cells* monophialidic (15–) 24–34 × 3.6–5 μm (av. = 27.6 × 4 µm, n = 20), subcylindrical, light brown, narrowing below the collarette. Collarettes light brown, funnel-shaped, 2.2–4.9 μm at the opening, 0.8–1.1 μm at deep. *Conidia* accumulating at the heads white, 23–32 × 2.5–3.2 μm (av. = 26.1 × 2.9 µm, n = 30), hyaline, unicellular, thin-walled, smooth, abundant guttulate, falcate to subclavate, rarely straight, narrowly rounded at the both ends, with 6–11 μm long hair-like appendages at both ends, smooth (Fig. 2).

##### Culture characteristics

Conidia germinating on PDA within 12 h. *Colonies* growing on PDA, reaching 20–30 mm diam. after 3 weeks at 28°C, circular, white to pale grey mycelium with hyaline margin, centre lightly raised, pale brown to dark brown in reverse, with smooth margin.

##### Material examined

CHINA, Jiangxi Province, Jian, Dingjiang, on submerged wood in a stream, 9 April 2018, J.E. Huang (HFJAU 3175, **Holotype**); ex-type living culture (JAUCC 2824).

#### Etymology

'*jiangxiensis*' refering to the host location, Jiangxi Province, where the holotype was collected.

#### Notes

*Dictyochaetajiangxiensis* is a distinct species in the genus as supported by molecular phylogenetic analysis and it clusters with *D.brevis*, but the latter has smaller conidia (7.5–11.4 µm long, 2.0–2.9 μm wide; Lin et al. (2019)). We found that there was 8% nucleotide difference of ITS sequences and about 2% nucleotide difference between the LSU sequences of *Dictyochaetajiangxiensis* sp. nov. JAUCC2824 and *Dictyochaetabrevis* MFLU 19-0216. Morphologically, *D.jiangxiensis* matches *Dictyochaeta* well, especially the setae surrounded by several conidiophores and conidia with setulae at both ends. *D.jiangxiensis* is similar to *D.fuegiana* (*Chaetosphaeriafuegiana*), *D.occidentalis*, *C.siamensis* (*Dictyochaetasiamensis*) and *C.lignicola* (*Dictyochaetalignicola*) in having multi-septae and a single phialide at the apex, subcylindrical conidiogenous cells with funnel-shaped collarette and guttulate conidia with hair-like appendages. However, *D.fuegiana* (*Chaet.fuegiana*) has smaller conidia (15–23 × 2–2.5 μm) without hair-like appendages at both ends (Spegazzini 1923). *C.lignicola* also has smaller conidia (13–15 μm long, 4.5–5.5 μm wide) and has no setae (Luo et al. 2019). *D.occidentalis* has wider conidia (24–32 × 3–4 μm; Whitton et al. (2000)) with degenerated appendages. *C.siamensis* has mono- or polyphialidic conidiogenous cells and samller conidia (8–17 × 2–5 μm; Tibpromma et al. (2018)).

## Analysis

### Phylogenetic analyses

**Figure 1.**
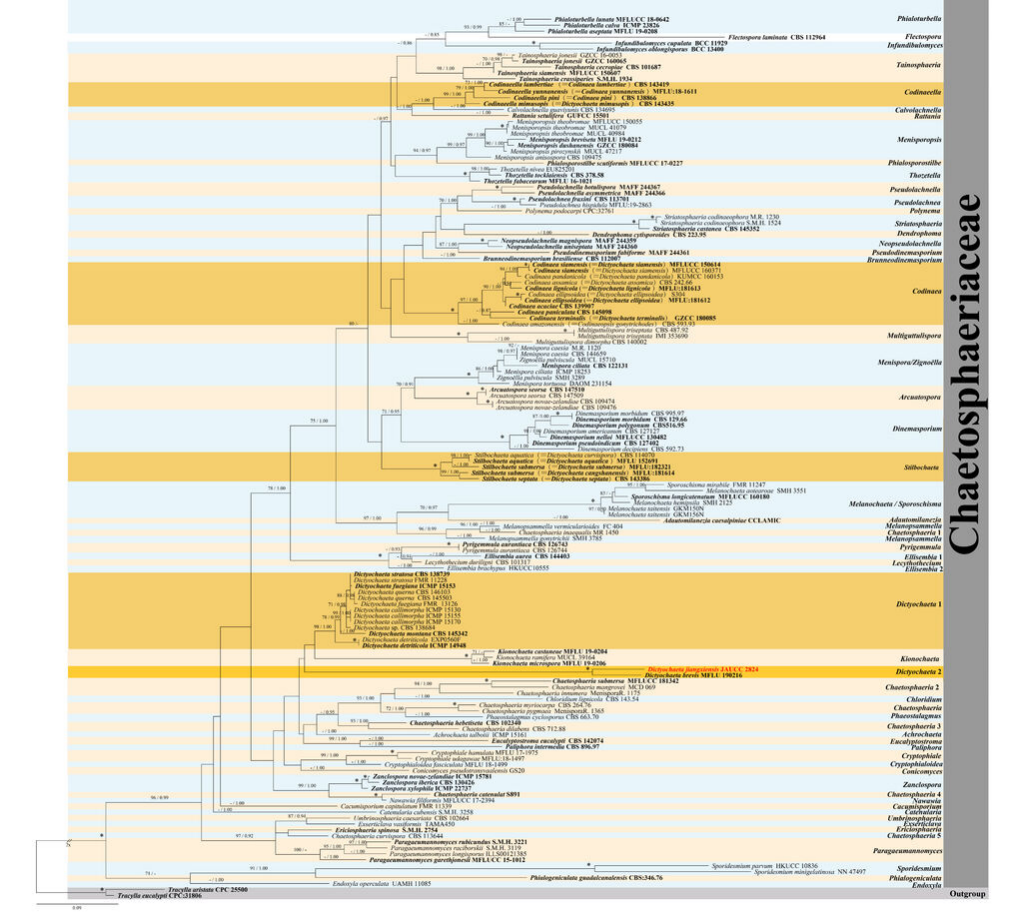
Phylogenetic tree based on combined ITS, LSU sequences of most taxa of the Chaetosphaeriaceae. Species name given in bold red is a new taxon in this study; species name given in bold indicates a type strain, respectively. Asterisk (*) indicates branches with MLBS = 100% and PP value = 1.0. The ML bootstrap support values and Bayesian posterior probabilities are given above the branches (MLBS/BPP). The tree is rooted to *Tracyllaaristata* CPC 25500 and *Tracyllaeucalypti* CPC:31806.

**Figure 2.**
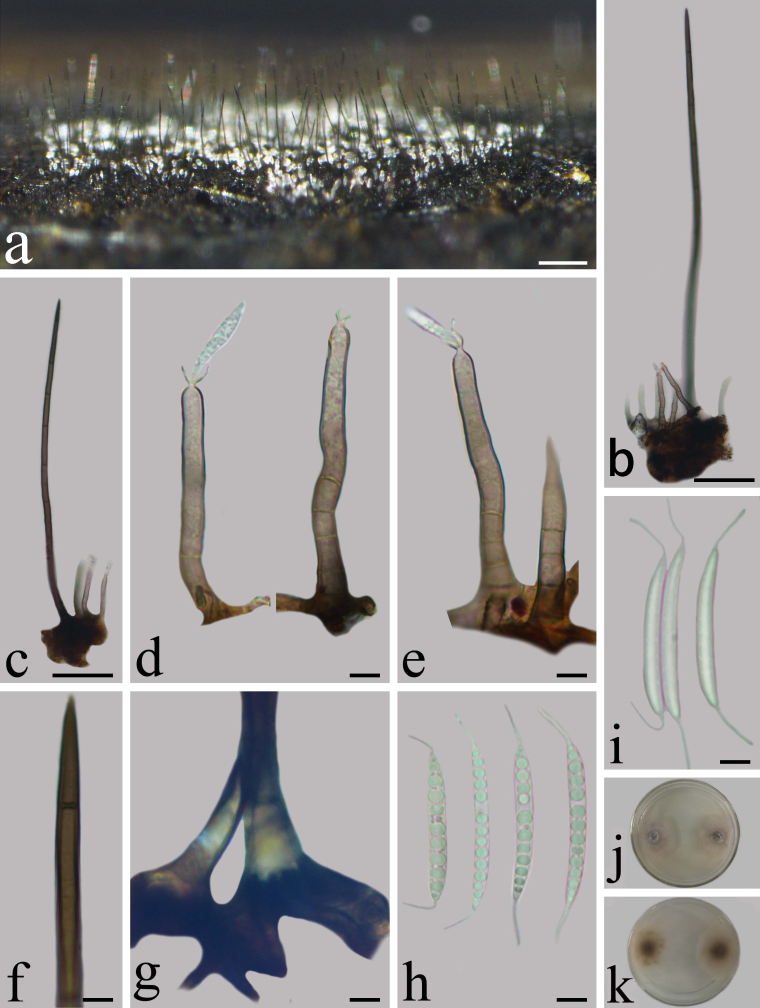
*Dictyochaetajiangxiensis* (HFJAU 3175, holotype). **a** Colonies on submerged wood; **b, c** Setae and conidiophores; **d, e** Conidiophores and phialides with a developing conidia; **f, g** Apex and base of setae; **h, i** Conidia; **j, k** Colony on PDA from above and below. Scale bars: a = 200 μm, b, c = 50 μm, d–h = 5 μm.

Based on ITS and LSU, a multi-locus phylogenetic tree was established to demonstrate the relationships between the new species and related taxa in Chaetosphaeriaceae (Fig. 1). The alignment has 1767 characters (including alignment gaps), with 715 characters for ITS and 1052 characters for LSU. The ML analysis result showed coincident topology with BI. Fig. 1 shows the ML tree based on the combined dataset, along with the fully supported bootstrap values and Bayesian posterior probabilities. All phylogenetic trees were similar in topologies.

The new species *Dictyochaetajiangxiensis*, together with *Dictyochaetabrevis* MFLU 19-0216, formed a well-supported clade (BPP = 1.00; MLBS = 100%), but formed a separate branch and there were obvious differences between them. By comparing the ITS and LSU sequences of *Dictyochaetajiangxiensis* sp. nov. JAUCC2824 and *Dictyochaetabrevis* MFLU 19-0216 respectively in NCBI, we found that there was 8% nucleotide difference of ITS sequences and about 2% nucleotide difference between the LSU sequences of *Dictyochaetajiangxiensis* sp. nov JAUCC2824 and *Dictyochaetabrevis* MFLU 19-0216.

## Discussion

Freshwater fungi refer to the fungi that rely on the freshwater habitats for the whole life cycle or part of the life cycle. Phylogenetic studies on freshwater ascomycetes have shown that some species cluster with terrestrial ascomycete lineages, while others cluster with exclusive aquatic lineages (Raja et al. 2018). However, Shearer (1993) defined freshwater fungi as “fungi that must rely on the freshwater environment to complete their life cycle”. The concept of aquatic fungi in a broad sense was adopted in this study. Chaetosphaeriaceae is a huge and diverse group with overwhelmingly phialidic fungi and some members of Chaetosphaeriaceae possess known teleomorphs. The family has a world-wide distribution. They are predominantly isolated from soil and plant debris, some are endophytic and have been isolated from herbaceous plants (Hughes and Kendrick 1968, Réblová 2004, Fernández and Huhndorf 2005, Huhndorf and Fernández 2005, Crous et al. 2012, Yang et al. 2018, Lin et al. 2019, Luo et al. 2019). In this study, the new *Dictyochaeta* species in the family Chaetosphaeriaceae was isolated from a freshwater environment. Most known species in *Dictyochaeta* were reported from rotting parts of plants, such as decaying leaf, bark or stem and submerged wood, partly from soil. Previously, *Dictyochaeta* and *Codinaea* were hard to demarcate, not only because of their morphological and ecological similarities, but their closely-related phylogenetic relationship. The difference between *Dictyochaeta* and *Codinaea* lies in the presence or absence of setae. The taxonomy of these fungi has relied mainly on morphological criteria. However, it is hard to treat setae as a criterion for identification as setae are always irregular amongst these similar taxa as mentioned above. *Codinaea* was introduced to accommodate a single species, *C.aristata*. Since then, the type species of *Codinaea* has become a taxonomic bottleneck. This species has not been recorded in any literature since its initial description. The holotype material and molecular data could not be traced. That is why the phylogenetic statuses of the *Dictyochaeta*-like fungi are still ambiguous. Hughes and Kendrick (1968) made an attempt at using the name *Dictyochaeta* instead of *Codinaea* on account of the principle of priority and suggested to adopt the name *Codinaea* as the type material for *D.fuegiana* of *Dictyochaeta*. Since Gamundí et al. (1977) redescribed *D.fuegiana* from fresh material, *Dictyochaeta* became a precedently used name. Crous et al. (2018) accepted this treatment. Simultaneously, the name *Codinaea* was suggested to be treated as the type material for *D.fuegiana* of *Dictyochaeta* by Hughes and Kendrick (1968), but this view has great limitations. Shortage of abundant original descriptions and loss of the type material of *Codinaea* were the factors (Liu et al. 2016) which indicated that the molecular phylogeny of *Dictyochaeta* has not been solved due to the small number of sequences in GenBank. The species, thus, need recollecting, epitypifying and sequencing to establish which morphological characters are of taxonomic significance and generic boundaries.

Recently, *Dictyochaeta* has still not been classified as monophyletic even though most *Dictyochaeta*-like and *Codinaea*-like species were re-assessed and recognised as five genera: *Codinaeella*, *Nimesporella*, *Stilbochaeta*, *Tainosphaeriella* and *Xyladelphia* (Réblová et al. 2021b). Réblová et al. (2021b) indicated *Codinaea* is a highly polyphyletic taxon unrelated to *Dictyochaeta* and that its original delimitation, based on a single morphotype of *C.aristata*, is too narrow and unsustainable and they emphasise the importance of combination of microscopic morphological characters developed in culture and under a natural substrate for identification. In our analysis, the phylogenetic position of some taxa, such as *Zignoëlla* and *Menispora*, are unclear in the tree with low BS and PP values. *Kionochaeta* formed a sister clade with *Dictyochaeta* and they were clustered together, but with low BS and PP values. Quite a number of species have not been adopted to establish the phylogenetic tree on account of the absence of molecular data and type materials. The molecular database and materials of Chaetosphaeriaceae species needs to be supplemented and improved.

## Supplementary Material

XML Treatment for Dictyochaeta jiangxiensis

## Figures and Tables

**Table 1.** Strains used in this study and their GenBank numbers. Note: Type strains are in bold. The underlined species indicated the new taxa in this study. The sequences of new species are indicated as underlined and unavailable sequences in GenBank are indicated by hyphen "-".

**Species**	**Strain number**	**GenBank accession numbers**	
		**ITS**	**LSU**
*Achrochaetatalbotii*	ICMP 15161	MT454480	MT454495
***Adautomilaneziacaesalpiniae***	CCLAMIC	KX821777	KU170671
*Arcuatosporanovae-zelandiae*	CBS 109474	MW984569	MW984552
*Arcuatosporanovae-zelandiae*	CBS 109476	MW984570	MW984553
*Arcuatosporaseorsa*	CBS 147509	MW984571	MW984554
***Arcuatosporaseorsa***	CBS 147510	MW984572	MW984555
*Brunneodinemasporiumbrasiliense*	CBS 112007	JQ889272	JQ889288
*Cacumisporiumcapitulatum*	FMR 11339	HF677176	——
*Calvolachnellaguaviyunis*	CBS 134695	KJ834524	KJ834525
*Catenulariacubensis*	S.M.H. 3258	MW987826	——
***Chaetosphaeriacatenulat***	S891	——	MK835838
*Chaetosphaeriacurvispora*	CBS 113644	——	GU180636
*Chaetosphaeriadilabens*	CBS 712.88	AF178557	AF178557
***Chaetosphaeriahebetiseta***	CBS 102340	AF178549	AF178549
*Chaetosphaeriainaequalis*	MR 1450	AF178564	AF178564
*Chaetosphaeriainnumera*	MenisporaR. 1175	AF178551	AF178551
*Chaetosphaeriamangrovei*	MCD 069	MG813821	MG813820
*Chaetosphaeriamyriocarpa*	CBS 264.76	AF178552	AF178552
*Chaetosphaeriapygmaea*	MenisporaR. 1365	AF178545	AF178545
***Chaetosphaeriasubmersa***	MFLUCC 181342	MK828634	MK835835
*Chloridiumlignicola*	CBS 143.54	AF178544	AF178544
***Codinaeaacaciae***	CBS 139907	KR476732	——
***Codinaealambertiae***	CBS 143419	MG386052	MG386105
***Codinaeapaniculata***	CBS 145098	MT118230	MT118201
***Codinaeapini***	CBS 138866	KP004465	KP004493
***Codinaeayunnanensis***	MFLU:18-1611	MK828623	MK835823
*Codinaeopsisgonytrichodes*	CBS 593.93	AF178556	AF178556
*Conicomycespseudotransvaalensis*	GS20	LC001710	LC001708
*Cryptophialehamulata*	MFLU 17-1975	——	MG386756
*Cryptophialeudagawae*	MFLU:18-1497	MH758198	MH758211
*Cryptophialoideafasciculata*	MFLU 18-1499	MH758195	MH758208
*Dendrophomacytisporoides*	CBS 223.95	JQ889273	JQ889289
*Dictyochaetaaquatica*	MFLU 152691	MH476572	MH476569
*Dictyochaetaassamica*	CBS 242.66	MH858788	MH870426
*Dictyochaetabrevis*	MFLU 190216	MN104614	MN104625
*Dictyochaetacallimorpha*	ICMP 15155	MT454484	MT454499
*Dictyochaetacallimorpha*	ICMP 15170	MT454485	MT454500
*Dictyochaetacallimorpha*	ICMP 15130	MT454483	MT454498
***Dictyochaetacangshanensis***	MFLU:181614	MK828632	MK835832
*Dictyochaetacurvispora*	CBS 114070	MH862954	——
***Dictyochaetadetriticola***	ICMP 14948	MT454486	MT454501
*Dictyochaetadetriticola*	EXP0560F	DQ914666	——
***Dictyochaetaellipsoidea***	MFLU:181612	MK828628	MK835828
*Dictyochaetaellipsoidea*	S304	MK828627	MK835827
*Dictyochaetafuegiana*	ICMP 15153	MT454487	EF063574
*Dictyochaetafuegiana*	FMR_13126	KY853440	KY853500
***Dictyochaetajiangxiensis***	JAUCC 2824	MN619652	MN607224
***Dictyochaetalignicola***	MFLU:181613	MK828630	MK835830
***Dictyochaetamimusopis***	CBS 143435	MH107888	MH107935
***Dictyochaetamontana***	CBS 145342	MT454488	MT454502
*Dictyochaetapandanicola*	KUMCC 160153	MH388338	MH376710
*Dictyochaetaquerna*	CBS 146103	MT454490	MT454504
*Dictyochaetaquerna*	CBS 145503	MT454489	MT454503
***Dictyochaetaseptata***	CBS 143386	MH107889	MH107936
*Dictyochaetasiamensis*	MFLUCC 160371	MH388339	MH376711
***Dictyochaetasiamensis***	MFLUCC 150614	KX609955	KX609952
*Dictyochaetasimplex*	CBS 966.69	AF178559	AF178559
*Dictyochaetasimplex*	MFLU 190202	MN104609	MN104620
*Dictyochaeta* sp.	CBS 138684	MT454493	MT454507
***Dictyochaetastratosa***	CBS 138739	MT454491	MT454505
*Dictyochaetastratosa*	FMR 11228	MT454492	MT454506
***Dictyochaetasubmersa***	MFLU:182321	MK828631	MK835831
***Dictyochaetaterminalis***	GZCC 180085	MN104613	MN104624
***Dinemasporiumamericanum***	CBS 127127	JQ889274	JQ889290
*Dinemasporiumdecipiens*	CBS 592.73	JQ889275	JQ889291
***Dinemasporiummorbidum***	CBS 129.66	JQ889280	JQ889296
*Dinemasporiummorbidum*	CBS 995.97	JQ889281	JQ889297
***Dinemasporiumnelloi***	MFLUCC 130482	KP711358	KP711363
***Dinemasporiumpolygonum***	CBS516.95	JQ889276	JQ889292
***Dinemasporiumpseudoindicum***	CBS 127402	JQ889277	JQ889293
***Ellisembiaaurea***	CBS 144403	MH836375	MH836376
*Ellisembiabrachypus*	HKUCC10555	——	DQ408563
*Endoxylaoperculata*	UAMH 11085	——	JX460992
***Ericiosphaeriaspinosa***	S.M.H. 2754	MW984575	AF466079
***Eucalyptostromaeucalypti***	CBS 142074	KY173408	KY173500
*Exserticlavavasiformis*	TAMA450	——	AB753846
***Flectosporalaminata***	CBS 112964	MW984576	MW984558
***Infundibulomycescupulata***	BCC 11929	EF113976	EF113979
***Infundibulomycesoblongisporus***	BCC 13400	EF113977	EF113980
***Kionochaetacastaneae***	MFLU 19-0204	MN104610	MN104621
***Kionochaetamicrospora***	MFLU 19-0206	MN104607	MN104618
*Kionochaetaramifera*	MUCL 39164	MW144421	MW144404
*Lecythotheciumduriligni*	CBS 101317	——	AF261071
*Melanochaetaaotearoae*	SMH 3551	——	AF466082
*Melanochaetahemipsila*	SMH 2125	——	AY346292
*Melanochaetataitensis*	GKM156N	——	EU583220
*Melanochaetataitensis*	GKM150N	——	EU583219
*Melanopsammellagonytrichii*	SMH 3785	——	AF466085
*Melanopsammellavermicularioides*	FC 404	——	AF466087
*Menisporacaesia*	M.R. 1120	AF178543	——
*Menisporacaesia*	CBS 144659	MW984578	MW984560
*Menisporaciliata*	ICMP 18253	——	GU180637
***Menisporaciliata***	CBS 122131	EU488736	——
*Menisporatortuosa*	DAOM 231154	KT225527	AY544682
*Menisporopsisanisospora*	CBS 109475	MH862827	MH874421
***Menisporopsisbreviseta***	MFLU 19-0212	MN104612	MN104623
***Menisporopsisdushanensis***	GZCC 180084	MN104615	MN104626
*Menisporopsispirozynskii*	MUCL 47217	MW984579	MW984561
*Menisporopsistheobromae*	MFLUCC 150055	KX609957	KX609954
*Menisporopsistheobromae*	MUCL 41079	MW984580	MW984562
*Menisporopsistheobromae*	MUCL 40984	MW984581	MW984563
*Multiguttulisporadimorpha*	CBS 140002	MW984582	MW984564
*Multiguttulisporatriseptata*	CBS 487.92	MW984583	MW984565
*Multiguttulisporatriseptata*	IMI 353690	MW984584	MW984566
*Nawawiafiliformis*	MFLUCC 17-2394	MH758196	MH758209
***Neopseudolachnellamagnispora***	MAFF 244359	AB934066	AB934042
***Neopseudolachnellauniseptata***	MAFF 244360	AB934067	AB934043
***Paliphoraintermedia***	CBS 896.97	MH862682	MH874289
***Paragaeumannomycesgarethjonesii***	MFLUCC 15-1012	KY212751	KY212759
*Paragaeumannomyceslongisporus*	ILLS00121385	MT118237	MT118211
*Paragaeumannomycesraciborskii*	S.M.H. 3119	AY906953	AY436402
***Paragaeumannomycesrubicundus***	S.M.H. 3221	MT118242	MT118224
*Phaeostalagmuscyclosporus*	CBS 663.70	MH859892	MH871680
***Phialogeniculataguadalcanalensis***	CBS:346.76	MH860986	MH872756
***Phialosporostilbescutiformis***	MFLUCC 17-0227	MH758194	MH758207
***Phialoturbellaaseptata***	MFLU 19-0208	MN104611	MN104622
***Phialoturbellacalva***	ICMP 23826	MW984585	MW984567
***Phialoturbellalunata***	MFLUCC 18-0642	MK828624	MK835824
*Polynemapodocarpi*	CPC:32761	MH327797	MH327833
***Pseudodinemasporiumfabiforme***	MAFF 244361	AB934068	AB934044
***Pseudolachneafraxini***	CBS 113701	JQ889287	JQ889301
*Pseudolachneahispidula*	MFLU:19-2863	MT185550	MT183515
***Pseudolachnellaasymmetrica***	MAFF 244366	AB934073	AB934049
***Pseudolachnellabotulispora***	MAFF 244367	AB934074	AB934050
***Pyrigemmulaaurantiaca***	CBS 126743	HM241692	HM241692
*Pyrigemmulaaurantiaca*	CBS 126744	HM241693	HM241693
***Rattaniasetulifera***	GUFCC 15501	GU191794	HM171322
*Sporidesmiumminigelatinosa*	NN 47497	——	DQ408567
*Sporidesmiumparvum*	HKUCC 10836	——	DQ408558
***Sporoschismalongicatenatum***	MFLUCC 160180	KX505871	KX358077
*Sporoschismamirabile*	FMR 11247	HF677174	HF677183
***Striatosphaeriacastanea***	CBS 145352	MT118244	MT118229
*Striatosphaeriacodinaeophora*	M.R. 1230	AF178546	AF178546
*Striatosphaeriacodinaeophora*	S.M.H. 1524	MT118245	AF466088
***Tainosphaeriacecropiae***	CBS 101687	MW984586	MW984568
***Tainosphaeriacrassiparies***	S.M.H. 1934	MW984587	AF466089
***Tainosphaeriajonesii***	GZCC 160065	KY026060	KY026057
*Tainosphaeriajonesii*	GZCC 16-0053	MN121305	KY026056
***Tainosphaeriasiamensis***	MFLUCC 150607	KX609956	KX609953
***Thozetellafabacearum***	MFLU 16-1021	KY212754	KY212762
*Thozetellanivea*	EU825201	EU825201	EU825200
***Thozetellatocklaiensis***	CBS 378.58	MH857817	MH869349
***Tracyllaaristata***	CPC 25500	KX306770	KX306795
***Tracyllaeucalypti***	CPC:31806	MH327810	MH327846
*Umbrinosphaeriacaesariata*	CBS 102664	——	AF261069
***Zanclosporaiberica***	CBS 130426	KY853480	KY853544
***Zanclosporanovae-zelandiae***	ICMP 15781	MW144429	MW144411
***Zanclosporaxylophila***	ICMP 22737	MW144437	MW144417
*Zignoëllapulviscula*	MUCL 15710	——	AF466090
*Zignoëllapulviscula*	SMH 3289	——	AF466091
